# Associations between sarcopenia and depression in middle-aged and older adults: the moderating effect of smoking

**DOI:** 10.1038/s41598-024-65343-3

**Published:** 2024-07-02

**Authors:** Feiyun Zhu, Jing Guo, Weijun Zheng

**Affiliations:** 1 School of Public Health, Zhejiang Chinese Medical University, Office 412, 548# Bingjiang District, Hangzhou, 310053 Zhejiang China; 2 Zhejiang Provincial Key Laboratory of Precision Diagnosis and Therapy for Major Gynecological Diseases, Women’s Hospital, Zhejiang University School of Medicine, Hangzhou, Zhejiang China

**Keywords:** Sarcopenia, Depression, CES-D score, Epidemiology, Epidemiology, Psychiatric disorders

## Abstract

The aim of this study were to estimate associations of sarcopenic status with depressive symptoms. We used mixed-effects linear model to estimate longitudinal association between sarcopenic status and rate of change in 10-item Center for Epidemiologic Studies Depression (CES-D) scores, and used Cox regression model to estimate the association between sarcopenic status and incident depression (CES-D ≥ 10). Stratification analyses were performed when the interactions between sarcopenic status and covariates were significant. A total of 6522 participants were ultimately included. After adjusting for covariates, participants with possible sarcopenia (β = 0.117; 95% CI 0.067 to 0.166; *P* < 0.001) and sarcopenia (*β*: 0.093; 95% CI 0.027–0.159;* P* < 0.001) had a faster increase in CES-D scores compared with normal individuals. Interactions between smoking and sarcopenic status were significant (*P*_interaction_ < 0.05). We found significantly positive associations of sarcopenic status with CES-D scores in nonsmokers, but not in current and past smokers. Besides, compared with normal participants, those with possible sarcopenia (HR 1.15; 95% CI 1.05 to 1.27) and sarcopenia (HR 1.28; 95% CI 1.12 to 1.46) (*P*_trend_ < 0.001) had elevated risks of incident depression. Sarcopenia is associated with a faster increase in CES-D scores and increased risks of depression among Chinese middle-aged and older adults. Stronger associations between sarcopenia and trajectory of CES-D scores were found in nonsmokers than in smokers.

## Introduction

Depression is a prevalent mental disorder that affects 280 million people globally^1^, accounting for 3.8% of the worldwide population, including 5.0% of adults and 5.7% of elderly adults ≥ 60 years^2^. Depression is expected to overtake diabetes as the most frequent debilitating disease in the world by 2030^3^. A meta-analysis has shown that more than one-third of older persons have the experience of depressive symptoms^4^. Some studies have shown that depression may lead to accelerated cellular aging^5^ and that depression may have lasting and adverse effects on cognitive function^6^. Depression also has linkage to premature death caused by other diseases and suicide^7^. Therefore, identification of modifiable risk factors for depression is expected for prevention.

Sarcopenia, a progressive and generalized muscle disease that involves muscle mass, muscle strength and physical function, is associated with a series of adverse health outcomes including falls, fractures, and mortality^8,9^. According to a meta-analysis, the prevalence of sarcopenia in people under the age of 60 ranged from 8 to 36% and in people over 60 from 10 to 27%; the prevalence of severe sarcopenia was found to be around 2% and 9%^10^. The association between sarcopenia and depression has been shown to be inconsistent in some previous studies^11–14^. Associations between sarcopenia and depression were significantly positive in a study in the Taiwan area^15^, but were nonsignificant from another study from South Korea^16^. According to the latest study, a healthy lifestyle was found to have a moderating effect on the association between sarcopenia and depression. However, a specific analysis of which factor has the greatest impact was not provided^17^. Moreover, associations between sarcopenia and rate of change in depressive symptoms have not been explored previously.

According to a previous longitudinal study, researchers found that smokers had a 2.36 times higher risk of developing sarcopenia compared to non-smokers, indicating smoking as a significant predictive factor for sarcopenia onset^2^; a review comprehensively summarized cellular models of cigarette smoke-induced skeletal muscle protein breakdown, Ref.^3^ demonstrating the significant impact of smoking on muscles. Additionally, in another longitudinal study, researchers found that smokers without baseline depression had a 20% higher likelihood of developing depression during follow-up compared to non-smokers^18^. Smoking, as a modifiable risk factor, needs to be comprehensively considered for its role in the association between sarcopenia and depression.

Based on the longitudinal data from China Health and Retirement Longitudinal Study (CHARLS) waves 1–4 (2011–2018), we aimed to examine associations of sarcopenia with trajectory of depressive symptoms and risks of incident depression among adults aged 45 years and older.

## Methods

### Study population

**Figure 1:**
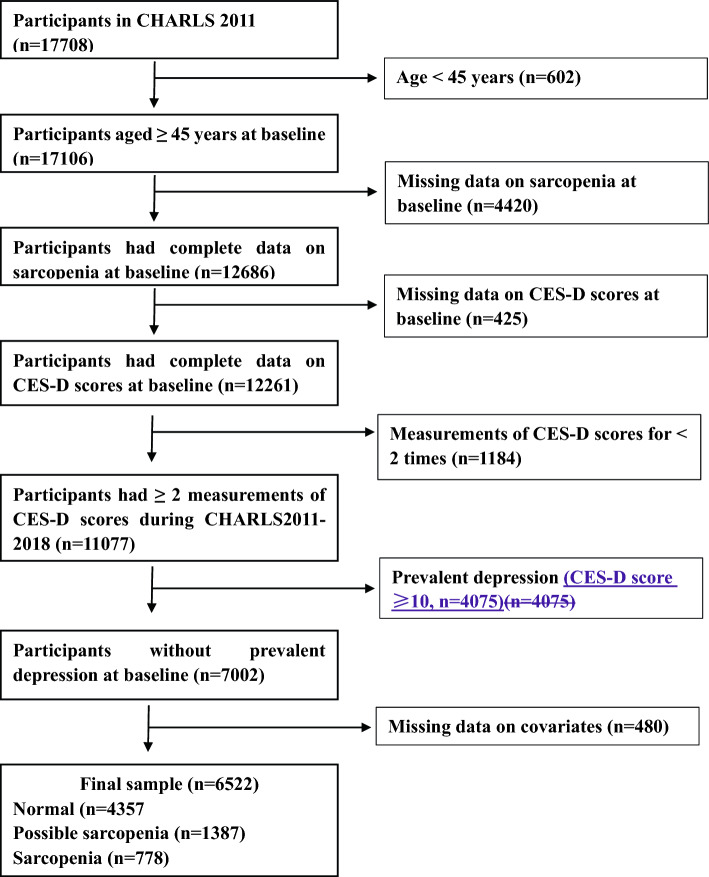
Flow chart of participant selection.

The CHARLS study is a nationally representative longitudinal survey which covers 150 county-level units in 28 Chinese provinces. Participants in CHARLS study were recruited in 2011 and were interviewed every two to three years to gather multifaceted information on health status, sociometric conditions, and lifestyles among adults aged 45 years and older. Investigative practices and implementation employed in CHARLS study have been described in detail previously^19^. Sarcopenia status and depressive symptoms were assessed during waves 1–3 (CHARLS 2011–2015) and waves 1–4 (CHARLS 2011–2018), respectively. As a result, we used eight-year data of waves 1–4 for data analyses and set CHARLS 2011 as the baseline (n = 17,708) to examine associations between sarcopenia status and subsequent rate of change in depressive symptoms and risks of incident depression. Participants who were eligible in the present study were those who aged 45 years and older at recruitment, had complete data on sarcopenia status at baseline, had no depression at baseline, had at least two measurements of CES-D scores during follow-up period, and had complete data on covariates. A total of 6522 participants were ultimately included in this study, of whom 4357 had no sarcopenia, 1387 had possible sarcopenia, and 778 had sarcopenia. The flow chart of participant selection is shown in (Fig. 1).

Ethical approval for all the CHARLS waves was granted from the Institutional Review Board at Peking University. The IRB approval number for the main household survey, including anthropometrics, is IRB00001052-11015. The study was conducted in accordance with the Declaration of Helsinki. All participants provided informed consent to take part in.

### Assessment of sarcopenia status

According to the criteria recommended by the Asian Working Group for Sarcopenia (AWGS) 2019 consensus, sarcopenia was assessed in terms of muscle strength, muscle mass, and physical function (19). Grip strength (kg) was measured when participants were asked to bend their elbows at a 90° angle and squeeze the dynamometer (YuejianTM WL-1000, Nantong Yuejian Physical Measuring Instruments Co., Ltd., China) with one hand as hard as they could for a few seconds^19^. Both left and right hands were separately measured for twice. The maximum value of records on grip strength was used. Low muscle strength was defined as less than 28 kg for men and 18 kg for women^20^. Appendicular skeletal muscle mass (ASM) was estimated by using the equation $ASM=0.193\times weight\left({\text{kg}}\right)+0.107\times height\left({\text{cm}}\right)-4.157\times gender\left(male=1, female=2\right)-0.037\times age\left({\text{years}}\right)-2.631,$ which has been proven to be validated in Chinese adults^21^. Muscle mass index (SMI) was then calculated as ASM divided by height in meters squared. In accordance with a previous study, a low muscle mass was defined as a SMI less than the 20th percentile in participants^22^, namely < 7.01 kg/m^2^ for males and < 5.28 kg/m^2^ for females in the present study. Limited physical function was characterized as gait speed < 1.0 m/s or five-time chair stand test ≥ 12 s. Gait speed was obtained when participants were asked to walk 2.5 m along a four-meter, non-carpeted walking course. Five-time chair stand test was performed when participants were instructed to keep arms folded across chest and stand up and sit down as fast as possible for five times.

Following to previous studies^20–23^, possible sarcopenia was determined by low muscle strength or five-time chair stand test ≥ 12 s, sarcopenia was defined as low muscle mass combined with low muscle strength or limited physical function, and severe sarcopenia was diagnosed by the detection of low muscle mass, low muscle strength, and limited physical function. Participants with severe sarcopenia were united into the sarcopenia group because only 144 (2.2%) individuals exhibited severe sarcopenia in this study. As a result, participants were divided into three groups: no sarcopenia, possible sarcopenia, and sarcopenia.

### Assessment of depressive symptoms and depression

Depressive symptoms were quantified with a ten-item Center for Epidemiologic Studies Depression Scale (CES-D)^24^ which has been widely used and proven to be reliable in older adults^25,26^. Participants were asked about the number of days they experienced a specific depressive symptom during the previous week. Each item was assigned 0 (less than 1 day), 1 (1–2 days), 2 (3–4 days), and 3 points (5–7 days) according to the reported days. The overall CES-D scores were calculated by summing scores of each item (ranging from 0 to 30), with higher scores indicating severer symptoms. Participants who had CES-D scores ≥ 10 were regarded as those with depression^27^.

### Covariates

Covariates were determined following to previous studies^11^. Participants were interviewed face-to-face to obtain information on sociodemographic characteristics including age, sex, education, and marital status, and lifestyle factors including smoking and alcohol consumption status with structured questionnaires. Educational levels were categorized into four groups of illiterate, primary, secondary, high school and above. Marital status was divided into three groups of married/cohabiting, divorced/separated/widowed, and unmarried. Smoking status was divided into nonsmoking, current smoking, and past smoking. The frequency of alcohol consumption (drinks per day) was used to quantify drinking levels. Participants with disability were those who reported some difficulties in any of six abilities of daily living including getting dressed, bathing or showering, eating, getting in and out of bed, using the toilet, and regulating urine and feces. Body weight and height were measured with standardized protocols. Body mass index (BMI) was calculated as weight in kilograms divided by height in meters squared. Comorbidities were assessed by summing numbers of self-reported chronic diseases including hypertension, diabetes, lung disease, heart disease, stroke and arthritis.

### Statistical analysis

The characteristics of the participants were expressed as mean ± S.D. (standard deviation), frequency and percentage (categorical variables). Characteristics of participants across different sarcopenic groups were compared by using methods of Chi-square test for categorical variables and analysis of variance for continuous variables. Baseline data was used to analyze cross-sectional associations between sarcopenic status and CES-D scores with linear regression models. Longitudinal associations between baseline sarcopenic status and subsequent rate of change in CES-D scores were examined with mixed-effects linear regression models, where CES-D scores were used as dependent variables and sarcopenic status, time intervals between follow-up visits and baseline, and an interaction term of “sarcopenic status × time” were used as independent variables. A significant interaction indicated differential rate of change in CES-D scores as a function of sarcopenic status. To examine potential effects by each covariate on association results, a three-way interaction of “sarcopenic status × time × covariate” was added in the mixed-effects model and tested with Wald test. We found that smoking showed a significant three-way interaction (*P*_interaction_ < 0.05), and further stratified data analyses by smoking status.

Associations of sarcopenic status and risks of incident depression were examined by using Cox regression model. Follow-up time was years from baseline to the occurrence of incident depression or the last visit (which ever came first). A method of Schofield residuals was applied to test proportional hazard assumption of Cox regression model. For association analyses in mixed-effects model and Cox regression model, Model 1 was a crude model, Model 2 was adjusted for age and sex, and Model 3 was additionally adjusted for educational levels, marital status, smoking, drinking, BMI, comorbidities, and disability.

All data analyses were performed with R (version 4.2.1). Two-sided *P* values < 0.05 were regarded as statistically significant.

## Results

Baseline characteristics of participants are shown in Table 1. 6522 participants had a mean age of 57.94 (SD = 8.82) years, of which 3380 (51.82%) were males. Compared with participants at the normal group, those with possible sarcopenia and sarcopenia were more likely to be older, to be males, to have lower levels of education, to be separated/divorced/widowed, to be smokers, to have disability in daily living, to have less drinking, lower BMI, more comorbidities, and higher CES-D scores. In the present study, there were 2624 participants (accounting for 40.2%, 2624/6522) developed incident depression, with a mean follow-up time of 5.42 years.

**Table 1:** Baseline characteristics of participants (CHARLS 2011).

Characteristics	Total (n = 6522)	Sarcopenic status			*P* values
		Normal (n = 4357)	Possible sarcopenia (n = 1387)	Sarcopenia (n = 778)	
Age (years), mean (SD)	57.94 (8.82)	55.69 (7.59)	59.51 (8.83)	67.71 (7.96)	** < 0.001**
Sex, n (%)					** < 0.001**
Male	3380 (51.82%)	2353 (54.01%)	603 (43.48%)	424 (54.50%)	
Female	3142 (48.18%)	2004 (45.99%)	784 (56.52%)	354 (45.50%)	
Education, n (%)					** < 0.001**
Illiterate	1517 (23.26%)	790 (18.13%)	416 (29.99%)	311 (39.97%)	
Primary school	2595 (39.79%)	1670 (38.33%)	590 (42.54%)	335 (43.06%)	
Middle school	1523 (23.35%)	1179 (27.06%)	253 (18.24%)	91 (11.70%)	
High school and above	887 (13.60%)	718 (16.48%)	128 (9.23%)	41 (5.27%)	
Marital status, n (%)					** < 0.001**
Married/partnered	5928 (90.89%)	4073 (93.48%)	1240 (89.40%)	615 (79.05%)	
Separated/divorced/widowed	561 (8.60%)	267 (6.13%)	142 (10.24%)	152 (19.54%)	
Never married	33 (0.51%)	17 (0.39%)	5 (0.36%)	11 (1.41%)	
Smoking, n (%)					** < 0.001**
Never	3816 (58.51%)	2518 (57.79%)	896 (64.60%)	402 (51.67%)	
Past	600 (9.20%)	402 (9.23%)	118 (8.51%)	80 (10.28%)	
Current	2106 (32.29%)	1437 (32.98%)	373 (26.89%)	296 (38.05%)	
Drinking (drinks/day), mean (SD)	1.47 (0.72)	1.50 (0.73)	1.36 (0.66)	1.46 (0.75)	** < 0.001**
BMI (kg/m^2^), mean (SD)	24.80 (38.93)	25.43 (40.41)	24.86 (3.71)	21.20 (59.36)	**0.021**
Comorbidities, mean (SD)	0.74 (0.89)	0.69 (0.86)	0.93 (0.98)	0.67 (0.80)	** < 0.001**
Disability, n (%)					** < 0.001**
No	6024 (92.36%)	4117 (94.49%)	1212 (87.38%)	695 (89.33%)	
Yes	498 (7.64%)	240 (5.51%)	175 (12.62%)	83 (10.67%)	
CES-D score, mean (SD)	4.39 (2.79)	4.23 (2.77)	4.65 (2.82)	4.81 (2.83)	** < 0.001**

*BMI* body mass index, *CES-D* the Center for Epidemiological Studies-Depression, *n* frequency, *%* proportion, *SD* standard deviation.

Values in bold mean statistically significant (*P* < 0.05).

Cross-sectional associations between sarcopenic status and CES-D scores were shown in Table 2. We found that compared with participants who were normal, those with possible sarcopenia and sarcopenia had significantly higher CES-D scores in fully adjusted Model 3 (*P*_trend_ < 0.001). Positive associations were also found in Model 1 and Model 2.

**Table 2:** Cross-sectional associations between sarcopenia status and CES-D scores with linear regression models (CHARLS 2011).

Sarcopenic status	No. of participants	Model 1	Model 2	Model 3
		β (95% CI)^a^	β (95% CI)^b^	β (95% CI)^c^
Normal	4357	Reference	Reference	Reference
Possible sarcopenia	1387	0.421 (0.253, 0.589)	0.336 (0.164, 0.508)	0.200 (0.029, 0.371)
Sarcopenia	778	0.585 (0.372, 0.797)	0.453 (0.217, 0.688)	0.442 (0.207, 0.677)
*P*_trend_		< 0.001	< 0.001	< 0.001

*β* coefficient, *CI* confidence interval.

^a^Model 1 was a crude model.

^b^Model 2 was adjusted for age and sex.

^c^Model 3 was adjusted for covariates in Model 2 plus education, marital status, drinking, smoking, BMI, self-care capacity, comorbidities.

Longitudinal associations between sarcopenic status and trajectory of CES-D scores were shown in Table 3. Compared with participants in normal group, individuals in groups of possible sarcopenia [coefficients (β) = 0.117, 95% confidence interval (CI) 0.067 to 0.166] and sarcopenia (β = 0.093, 95% CI 0.027 to 0.159) had significantly faster increase in CES-D scores (*P*_trend_ < 0.001) in fully-adjusted Model 3. Similar results were found in Model 1 and Model 2. Besides, significantly positive associations between sarcopenic status and rate of change in CES-D scores were found in nonsmokers but not in past smokers or current smokers (*P* > 0.05).

**Table 3:** Longitudinal associations between sarcopenia status and trajectory of CES-D scores with mixed-effects models (CHARLS 2011–2018).

Subpopulation	No. of participants	Model 1	Model 2	Model 3
		β (95% CI)^a^	β (95% CI)^b^	β (95% CI)^c^
All				
Normal × time	4357	Reference	Reference	Reference
Possible sarcopenia × time	1387	**0.116 (0.066, 0.166)**	**0.117 (0.067, 0.166)**	**0.117 (0.067, 0.166)**
Sarcopenia × time	778	**0.090 (0.024, 0.157)**	**0.093 (0.026, 0.159)**	**0.093 (0.027, 0.159)**
*P*_trend_		< 0.001	< 0.001	< 0.001
Nonsmoking^d^				
Normal × time	2518	Reference	Reference	Reference
Possible sarcopenia × time	896	**0.129 (0.064, 0.194)**	**0.129 (0.065, 0.194)**	**0.129 (0.064, 0.194)**
Sarcopenia × time	402	**0.177 (0.082, 0.271)**	**0.177 (0.083, 0.272)**	**0.180 (0.086, 0.275)**
*P*_trend_		< 0.001	< 0.001	< 0.001
Past smoking^d^				
Normal × time	402	Reference	Reference	Reference
Possible sarcopenia × time	118	0.154 (− 0.005, 0.313)	0.156 (− 0.002, 0.315)	0.160 (0.001, 0.318)
Sarcopenia × time	80	− 0.128 (− 0.326,0.071)	− 0.121 (− 0.319, 0.078)	− 0.122 (− 0.320, 0.077)
*P*_trend_		0.842	0.892	0.891
Current smoking^d^				
Normal × time	1437	Reference	Reference	Reference
Possible sarcopenia × time	373	0.066 (− 0.024, 0.156)	0.064 (− 0.026, 0.154)	0.065 (− 0.025, 0.155)
Sarcopenia × time	296	0.040 (− 0.064, 0.144)	0.043 (− 0.061, 0.147)	0.042 (− 0.062, 0.146)
*P*_trend_		0.228	0.215	0.225

*β* coefficient, *CI* confidence interval.

^a^Model 1 was a crude model.

^b^Model 2 was adjusted for age and sex.

^c^Model 3 was adjusted for covariates in Model 2 plus education, marital status, drinking, smoking, BMI, self-care capacity, comorbidities.

^d^Smoking status was not adjusted in Model 3.

Values in bold mean statistically significant (*P* < 0.05).

As shown in Table 4, compared with participants without sarcopenic condition, those with possible sarcopenia [hazard ratio (HR) 1.15, 95% CI 1.05 to 1.27] and sarcopenia (HR 1.28, 95% CI 1.12 to 1.46) had an increased risk of incident depression of 15% and 28% (*P*_trend_ < 0.001), respectively. Similar results were found in different models which were adjusted for different covariates. The proportional hazard assumption was not violated based on the test of Schofield residuals (*P* = 0.247).

**Table 4:** Associations between sarcopenia status and risks of incident depression with Cox regression models (CHARLS 2011–2018).

Sarcopenic status	No. of cases/total number	Model 1	Model 2	Model 3
		HR (95% CI)^a^	HR (95% CI)^b^	HR (95% CI)^c^
Normal	1667/4357	Reference	Reference	Reference
Possible sarcopenia	629/1387	**1.29 (1.18, 1.42)**	**1.24 (1.13, 1.36)**	**1.15 (1.05, 1.27)**
Sarcopenia	328/778	**1.26 (1.12, 1.42)**	**1.27 (1.11, 1.45)**	**1.28 (1.12, 1.46)**
*P*_trend_		< 0.001	< 0.001	< 0.001

*β* coefficient, *CI* confidence interval, *HR* hazard ratio.

^a^Model 1 was a crude model.

^b^Model 2 was adjusted for age and sex,

^c^Model 3 was adjusted for covariates in Model 2 plus education, marital status, drinking, smoking, BMI, self-care capacity, comorbidities.

^d^Body mass index was not adjusted in Model 3.

Values in bold mean statistically significant (*P* < 0.05).

## Discussion

Results of this large, prospective cohort study of middle-aged and older adults over 45 years of age indicate that sarcopenia is associated with a higher increase overtime in CES-D scores and increased risks of incident depression. Besides, we found a stronger association between sarcopenia and trajectory of CES-D scores among the nonsmokers but not among smokers.

Our results showed that baseline sarcopenia status was cross-sectionally associated with severer depressive symptoms at baseline, longitudinally associated with an accelerated trajectory of depressive development, and prospectively associated with increased risks of incident depression during follow-up. Our findings were consistent with the results of several previous cross-sectional studies^14,15,28^ and a prospective study with a mean follow-up of 3.7 years^11^. Compared to the previous prospective study^11^, the present study had a longer follow-up period of 5.42 years. More importantly, associations between sarcopenia and rate of change in depressive symptoms have not been explored previously.

Potential mechanisms underlying the association between sarcopenia and depression include multiple factors, such as neurotrophic factors, chronic inflammation, oxidative stress, and physical activity^29^. Skeletal muscle tissue can produce neurotrophic factors, especially brain-derived neurotrophic factor (BDNF), which can increase synaptic plasticity and promote neurogenesis, especially in the hippocampus^30,31^. Neuroplasticity plays an important role in the development of depression, and the hippocampus is a key region of the brain associated with psychiatric disorders^30,31^. Inflammatory cytokines have been shown to affect sarcopenia and depression by interfering with metabolism or the activity of other cytokines, a common influence on both^29^. The primary cause of sarcopenia is lack of exercise^32^, while randomized controlled studies have confirmed the role of exercise in improving depressive symptoms^33^. A growing number of studies have shown that exercise enhances the expression of BDNF, improves antioxidant capacity, and has anti-inflammatory effects^34,35^.

The three-way interactions between smoking, sarcopenia, and CES-D scores were statistically significant, suggesting potential effects of smoking on the sarcopenia-depression association. Results of stratification analyses indicated that associations between sarcopenia and rate of change in CES-D scores were stronger in nonsmokers than in smokers. The corresponding mechanisms have not been elucidated until now. This discrepancy by smoking status could be explained by the following. First, participants with past- and current-smoking status had a smaller sample size compared to nonsmokers, which might induce a poorer statistically power. Second, evidence from previous epidemiological studies has indicated that smoking is a risk factor for depression^36,37^. As a result, effects of sarcopenia on depression might be offset by the detrimental effects of smoking among smokers. Besides, it has been proposed that hormonal dysregulation in hypothalamic–pituitary–adrenal (HPA) axis is a risk factor shared by sarcopenia and depression^30^. Results of animal studies indicate that chronic nicotine exposure affects the secretion of cortisol and activity of HPA-related monoamine neurotransmitter system^38^. Studies focusing on the biological mechanisms were needed in the future. It can be hypothesized that smoking, which affects the hormonal dysregulation in HPA axis, may mask the effect of sarcopenia on depressive symptoms. Comparatively, quantitative data from the CES-D score are more sensitive as an indication of outcome and can swiftly show changes in outcome during follow-up than depressed symptoms, which are evaluated based on the scale.

This study has several advantages. First, the CHARLS study has a big sample size, covers a large area, has strong population representation, and has a high degree of confidence in inferring the features of the complete population from the sample information. Second, the study made full use of database information, not only for the long follow-up period of the longitudinal study, but also for the findings of both dichotomous indicators of depression status and quantitative indicators of CES-D scores. We found consistent results with different methods of data analyses suggesting that our findings were robust. In addition, this is the first study in Asia to examine the association between sarcopenia status and the rate of change in CES-D scores, and finds the important result that the effect of sarcopenia status on the rate of change in CES-D scores is predominantly in the nonsmoking population, providing new ideas for depression prevention and early intervention.

However, there are some limitations in this study. First, rather than 6 m as stated in AWGS2019 criteria, the distance measured by the CHARLS database to measure step speed is 2.5 m. The length of the walk during the gait speed test, however, had no influence on the recorded gait speed, according to a comprehensive review of 48 studies that evaluated gait speed in older adults^39^. Consequently, a 2.5 m walk would be suitable for measuring the walking speed of senior Chinese citizens. Second, the present study, while informative, omitted an examination of the relationship between alterations in sarcopenia status and the rate of change in CES-D scores and depressive symptoms throughout the follow-up period.

In conclusion, sarcopenia was associated with an accelerated rate of increase in CES-D scores among Chinese adults aged 45 years and older, especially for nonsmokers. Sarcopenia was also associated with increased risks of incident depression. Sarcopenia may be used as an early sign for the development of depression. Interventional studies are warranted to confirm our findings in the future.

## Data Availability

The datasets used during the current study available from the corresponding author on reasonable request.
